# Emerging Variability in HIV-1 Genetics among Recently Infected Individuals in Yunnan, China

**DOI:** 10.1371/journal.pone.0060101

**Published:** 2013-03-26

**Authors:** Min Chen, Li Yang, Yanling Ma, Yingzhen Su, Chaojun Yang, Hongbing Luo, Huichao Chen, Ling Chen, Wenyun Yan, Yuhua Shi, Manhong Jia, Lin Lu

**Affiliations:** 1 Center for AIDS/STD Control and Prevention, Yunnan Center for Disease Control and Prevention, Kunming, Yunnan, China; 2 College of Public Health, Kunming Medical University, Kunming, Yunnan, China; Johns Hopkins School of Public Health, United States of America

## Abstract

**Background:**

Yunnan has the longest endured Human Immunodeficiency Virus-1 (HIV-1) epidemic in China, and the genetic diversity of HIV-1 constitutes an essential characteristic of molecular epidemiology in this region. To obtain a more comprehensive picture of the dynamic changes in Yunnan’s HIV-1 epidemic, a cross-sectional molecular epidemiological investigation was carried out among recently infected individuals.

**Methodology/Principal Findings:**

We sequenced partial *gag* (HXB2∶781–1861) and *env* (HXB2∶7002–7541) genes from 308 plasma samples of recently infected patients. With phylogenetic analysis, 130 specimens generated interpretable genotyping data. We found that the circulating genotypes included: CRF08_BC (40.8%), unique recombinant forms (URFs, 27.7%), CRF01_AE (18.5%), CRF07_BC (9.2%), subtype B (2.3%) and C (1.5%). CRF08_BC was the most common genotype, and was predominant in both intravenous drug users (IDUs) and heterosexually transmitted populations. CRF08_BC and CRF07_BC still predominated in eastern Yunnan, but CRF08_BC showed increasing prevalence in western Yunnan. Strikingly, the URFs raised dramatically in most regions of Yunnan. Seven different types of URFs were detected from 12 prefectures, suggesting that complicated and frequent recombination is a salient feature of Yunnan’s HIV-1 epidemic. Among URFs, two BC clusters with distinctive recombination patterns might be potential new CRF_BCs. CRF01_AE was no longer confined to the prefectures bordering Myanmar, and had spread to the eastern part of Yunnan, especially the capital city of Kunming, with a large number of infections in the transient population. The ratios of the main genotypes showed no statistical differences between infected IDUs and heterosexually transmitted infections.

**Conclusions/Significance:**

The changing patterns of the dominant HIV-1 genotypes in Yunnan indicate the complex evolving dynamic nature of the epidemic. Understanding new trends in molecular epidemiology of HIV-1 infection is critical for adjusting current prevention strategies and vaccine development in Yunnan.

## Introduction

Yunnan is located in southwestern China and has 16 prefectures and 129 counties. Geographically, Yunnan borders Myanmar, Laos and Vietnam and is situated along the drug trafficking routes that channel heroin into China from southeast Asia’s opium-producing “Golden Triangle” region. In 1989, the first Human Immumodeficiency Virus (HIV) epidemic in China was identified among intravenous drug users (IDUs) in Ruili County of Dehong Prefecture [1]. Since then, Yunnan has been one of the areas most affected by HIV in China. Furthermore, Yunnan serves as a primary entry point for the introduction of different HIV-1 genotypes into China. Thus, Yunnan is considered as an epicenter of the HIV-1 epidemic in China.

In the late 1980s, China’s first HIV-1 epidemic among IDUs was initiated by both subtype B (circulating in United States and Europe) and subtype B’ (the Thailand variant of subtype B, also referred to as Thai-B) strains imported from Thailand to Yunnan by drug trafficking through Myanmar [2], [3], [4]. In the early 1990s, another epidemic was introduced into Yunnan by Indian IDUs with subtype C strains (India C) [5], [6]. In 1994, subtype E strains (now referred to as CRF01_AE) were identified among commercial sex workers who had returned from Thailand to Yunnan [7]. Over time, the Thai-B subtype overtook the prototypical B subtype in frequency, increasing from 20% of all subtype B in 1990 to 90% in 1996 [2], [3], [4]. Thus, subtype B’ and subtype C strains co-circulated in Yunnan’s IDUs in the first half of 1990s. Meanwhile, intravenous drug injection was the predominant transmission route in the early HIV-1 epidemic of Yunnan [1], [8]. These circumstances provided the opportunity for recombination between subtype B’ and subtype C among the IDU population in Yunnan.

Though CRF07_BC and CRF08_BC were first detected from IDUs in Xinjiang Province and Guangxi Province in 1997, respectively [9], [10], it is likely that these two CRFs were initially established in Yunnan Province [11], [12] and spread through two different overland heroin trafficking routes: CRF07_BC northwestward to Xinjiang Province, and CRF08_BC eastward to Guangxi Province [13]. Sequence analysis suggests that CRF07_BC and CRF08_BC are closely related and may have evolved from a common parent [14]. However, understanding the origin of the two CRFs in Yunnan required additional retrospective molecular epidemiological investigations.

Subsequently,studies carried out from 2001 to 2006 showed that there were three major groups circulating in Yunnan: C/CRF07_BC/CRF08_BC, CRF01_AE and B [15], [16]; C/CRF07_BC/CRF08_BC viruses included C, CRF08_BC, CRF07_BC and new BC recombinants. Although distributed widely, CRF08_BC and CRF07_BC largely dominated in eastern Yunnan. However, new BC recombinants were also found in western Yunnan [15], [16], [17]. CRF01_AE was mainly distributed in the western region bordering Myanmar, in prefectures such as Dehong, Baoshan, Xishuangbanna and Puer [15], [16]. Statistically, C/CRF07_BC/CRF08_BC and CRF01_AE were dominant in IDUs and those with sexually transmitted infections, respectively, suggesting that HIV-1 subtype distribution was closely associated with risk factors.

HIV-1 molecular epidemiological surveillance in new infections is of prime importance for understanding the real-time dynamics of the HIV-1 epidemic in a region with complicated HIV-1 subtype prevalence, such as Yunnan. The “gold standard” method for measuring recent infections is a prospective cohort study. However, cohort studies are difficult and expensive to implement and prone to biases that could reduce the general applicability of the results. As an alternative method, the BED-capture enzyme immunoassay (CEIA) has been widely used to measure the proportion of HIV-1-specific IgGs among total IgGs in blood samples for the purpose of identifying infections that were acquired recently [18], [19]. Because the last large-scale HIV-1 molecular epidemiological study in Yunnan was done between 2002 and 2004 [16], we decided to evaluate the new HIV-1 subtype propagation. In this work, we performed a cross-sectional molecular epidemiological investigation among the recently infected population identified by BED-CEIA to uncover the new prevalent trends of HIV-1 genetic strains in Yunnan. We found that CRF08_BC was the most common genotype in Yunnan, and the distributions of the main HIV-1 genotypes showed no statistical differences between IDUs and the heterosexually transmitted population. Moreover, diverse recombinations emerged rapidly with the multiple genotypes co-existing in Yunnan.

## Materials and Methods

### Study Participants and Sample Collection

A total of 3034 HIV-1-positive plasma samples were identified between January 2009 and March 2009 from 461,661 individuals at local voluntary counseling and testing sites (VCT), medical institutions and sentinel surveillance sites in Yunnan Province. HIV-1 infection status was determined by an enzyme immunoassay and confirmed by Western blot assay (HIV BLOT 2.2, MP Diagnostics, Singapore). A total of 308 plasma samples identified as recent infections among 3034 HIV-1-positives were used for genotype analysis. Plasma was separated from whole blood and used to obtain HIV-1 RNA for subsequent analysis. All HIV tests are informed and voluntary. Written consents for HIV testing were obtained, in which the subjects agreed that if they have HIV, their samples can be used in the researches for the purpose of controlling and preventing HIV. Because no human experimentation and no investigation of host genetics were conducted, the authors were exempted from approval by the local ethical review committee at the Yunnan Centers for Disease Control and Prevention.

### HIV-1 Seroconversion Identified with BED-CEIA

BED-CEIA was performed according to the manufacturer’s instructions (Calypte HIV-1 BED incidence EIA, Calypte Biomedical Corporation, Portland, OR) [20]. Test specimens were initially run singly. If the normalized OD (ODn) was >1.2, the specimen was classified as being from a long-term seroconverter. Specimens with ODn <1.2 were tested again in triplicate to confirm the values. In confirmatory testing, specimens with ODn values <0.8 were considered to have undergone recent seroconversion.

### Amplification of HIV-1 Gene Fragments

Three hundred and eight plasma samples identified as recent infections were used for genotype analysis. Viral RNA was extracted from 140 µl of plasma by using the QIAamp Viral RNA Mini kit (Qiagen, Valencia, CA, United States) according to the manufacturer’s instructions, and was then subjected to nested polymerase chain reaction (PCR) to generate the fragments of the *gag* gene (HXB2∶781–1861) and the *env* gene (HXB2∶7002–7541). The gag fragment was amplified using One Step reverse transcription PCR (Takara, Dalian, China) with primers GAG-L (5′- TCGACGCAGGACTCGGCTTGC -3′) and GAG-E2 (5′- TCCAACAGCCCTTTTTCCTAGG -3′) in 25 µl reaction volume. Cycling conditions were as follows: 50°C for 30 min;94°C for 5 min, 55°C for 1 min,72°C for 2 min;94°C for 30 s,55°C for 45 s,72°C for 1 min 30 s,30 cycles;72°C for 10 min. The nested *gag* PCR was performed using 2×Taq PCR MasterMix (Tiangen, Beijing, China) with primers GUX (5′-AGGAGAGAGATGGGTGCGAGAGCGTC-3′) and GDX (5′- GGCTAGTTCCTCCTACTCCCTGACAT-3′) in 50 µl reaction volume. Cycling conditions: 94°C for 2 min, 55°C for 1 min,72°C for 1 min 30 s;94°C for 30 s,55°C for 45 s,72°C for 1 min 30 s,30 cycles;72°C for 10 min. The env fragment was amplified with primers 44F (5′-ACAGTRCARTGYACACATGG-3′) and 35R (5′-CACTTCTCCAATTGTCCITCA-3′) with cycling conditions: 50°C for 30 min;94°C for 2 min, 50°C for 1 min,72°C for 4 min;94°C for 30 s,55°C for 30 s,72°C for 2 min,30 cycles;72°C for 10 min. The nested *env* PCR was performed with primers 33F (5′- CTGTTIAATGGCAGICTAGC -3′) and 48R (5′- RATGGGAGGRGYATACAT -3′) in 50 µl reaction volume; Cycling conditions: 95°C for 2 min; 95°C for 15 s,55°C for 30 s,72°C 1 min 15 s, 5 cycles;95°C for 15 s,60°C for 30 s,72°C for 1 min,25 cycles; 72°C for 10 min. Each step was carried out with appropriate negative controls to detect possible contamination during the experiments. The generated products were analyzed using 1% agarose gel electrophoresis. Positive samples were sent to Biomed Co. (Beijing, China) for sequencing by using an ABI 3730XL automated DNA sequencer (Applied Biosystems, Carlsbad, USA) with sequencing primers: GUX/GDX (for *gag*) and 33F/48R (for *env*).

### Sequence Analysis

The contig assembly of sequences was performed using DNA sequence analysis software Sequencher 4.9 (Gene Codes, Ann Arbor, MI). The ClustalW Multiple alignment and manual editing were performed using Bio-Edit 7.0 software. The reference sequences were obtained from the NIH/NIAID-funded HIV Databases (http://hiv-web.lanl.gov/content/index), covering the major HIV-1 subtypes/CRFs. Some reference sequences which were previously characterized in China and countries surrounding Yunnan were included. Phylogenetic tree analyses were performed using the neighbor-joining method based on Kimura 2-parameter model with 1000 bootstrap replicates, using MEGA version 4.0 [21]. To demonstrate possible intertype mosaicism, candidate sequences were analyzed using the Recombination Identification Program (RIP; version 3.5.1) which is available at the HIV sequence database (http://hiv-web.lanl.gov) using the appropriate parameters. Similarity plot analyses (version 3.5.1; S. Ray, Johns Hopkins University, Baltimore, MD) were further performed using reference strains of subtype A1, subtype A2, subtype B, subtype C, subtype D, subtype F1, subtype J, CRF01_AE and CRF08_BC. The conditions are further descried in the figure legends.

### Geographic Distribution Analysis of HIV-1 Genotypes

HIV-1 genetic geographic distribution was analyzed with the public health geographic information system (PHGIS, China CDC). A Dot Density Map was used to display the distribution density of each HIV-1 genotype within Yunnan Province. For each genotype, the number of patients with the genotype in each prefecture was divided by 130 (the total number of patients in this study) to obtain the percentage of each genotype in each prefecture. When using PHGIS to map the data, one dot was defined as 0.025% of the population.

### Sequence Data

All the sequences obtained in this study were submitted to GenBank under accession numbers JX263434 to JX263661.

### Statistical Analysis

Statistical analyses for this report were conducted using the SPSS 17.0 statistical analysis software package (SPSS Inc. Chicago, IL). Categorical variables were compared using χ^2^. All tests were two-tailed and a p value <0.05 was considered statistically significant.

## Results

### Demographic Characteristics of Study Subjects

A total of 3034 newly diagnosed HIV-positive samples were collected from VCT, medical case reports and sentinel surveillance in Yunnan Province during the first quarter of 2009. Of these, 308 samples were identified as recent infections, all of which were used for the HIV-1 genetic analysis. For each sample, *gag* and *env* genes were amplified and sequenced. In total, we obtained 120 *gag* sequences and 108 *env* sequences. Combining the phylogenetic tree analysis of *gag* and *env*, we successfully genotyped 130 samples with a rate of 42.2% (130/308). The failure of amplification was most commonly due to poor storage and transportation conditions of samples. The constituent ratios of the 130 subjects genotyped and the total of 308 recently HIV-infected individuals showed no statistical differences by geographical area, gender, age, ethnicity, marital status and infection routes (Table 1).

**Table 1:** The distribution of subjects successfully genotyped.

			BED-CEIA positive subjects	Subjects with identified subtypes	χ2	*P*
**Total**		308		130		
**Prefecture**					4.460	0.996
Baoshan		13		7		
Chuxiong		13		4		
Dali		18		8		
Dehong		52		25		
Diqing		0		0		
Honghe		55		22		
Kunming		51		17		
Lijiang		3		1		
lincang		22		8		
Nujiang		1		1		
Puer		10		3		
Qujing		20		11		
Wenshan		29		13		
Xishuangbanna		8		5		
Yuxi		7		2		
Zhaotong		6		3		
**Gender**					0.280	0.599
Male		174		77		
Female		134		53		
**Age**					4.798	0.310
≤20		28		9		
21–30		131		52		
31–40		85		39		
41–50		41		13		
≥51		23		17		
**Race/ethnicity**					3.172	0.682
Han		212		94		
Dai		24		10		
Yi		14		3		
Hani		10		2		
Jingpo		9		6		
Others		39		15		
**Marital Status**					0.185	0.909
Unmarried		106		42		
Married		167		73		
Divoiced/Widowed		35		15		
**Infection Routes**					3.969	0.400
Heterosexual contact		225		100		
Intravenous drug injection		61		20		
Homosexual contact		7		3		
Maternal-neonatal transmission		5		5		
Unknown		10		2		

As shown in Table 1, among the 130 subjects, the ratio of males to females was 1∶0.69. The mean age was 33.9 years (range: 1–79 years); And 72.3% (94/130) of individuals were of Han ethnicity, and 27.7% of individuals were minority nationality, including Dai, Jingpo, Bai, Yi, Zhuang, Hani, Miao, Wa, Yao, Buyi, Hui and Menggu. Heterosexual transmission was the chief transmission route, accounting for 76.9% (100/130), and intravenous drug injection ranked second, accounting for 15.4% (20/130), while other transmission routes that included homosexual transmission, maternal-neonatal transmission and unknown routes, accounted for 7.7% (10/130) of infections.

### HIV-1 Genotype Analysis in Recent Infections

**Figure 1:**
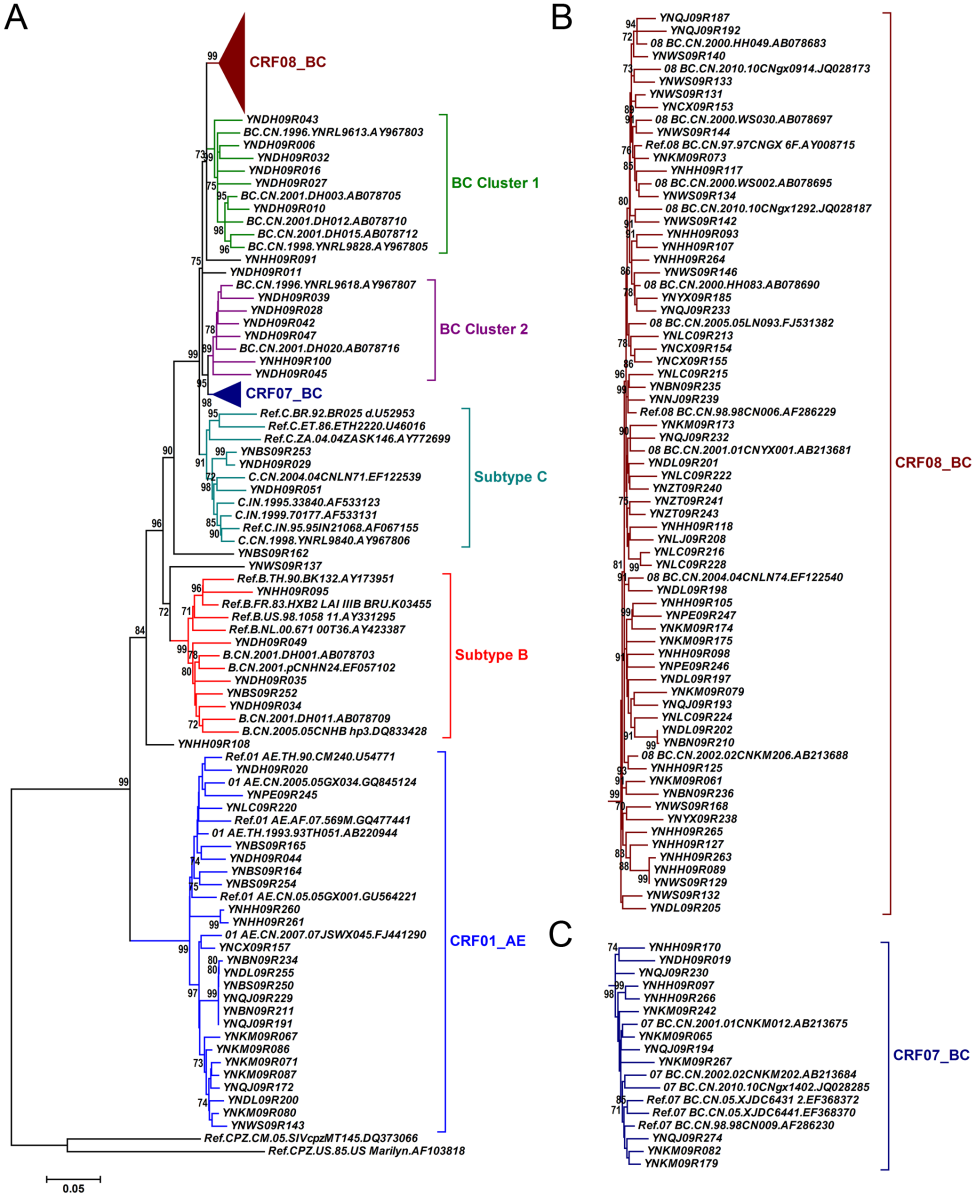
Neighbor-joining phylogenetic tree of partial *gag* gene from recently infected individuals confirmed by BED-CEIA. A, Neighbor-joining phylogenetic tree for 120 *gag* sequences and relative reference sequences. B and C, CRF08_BC and CRF07_BC clusters from the phylogenetic tree shown in A. The scale bar indicates 5% nucleotide sequence divergence. Values on the branches represent the percentage of 1000 bootstrap replicates and bootstrap values over 70% are shown in the tree.

**Figure 2:**
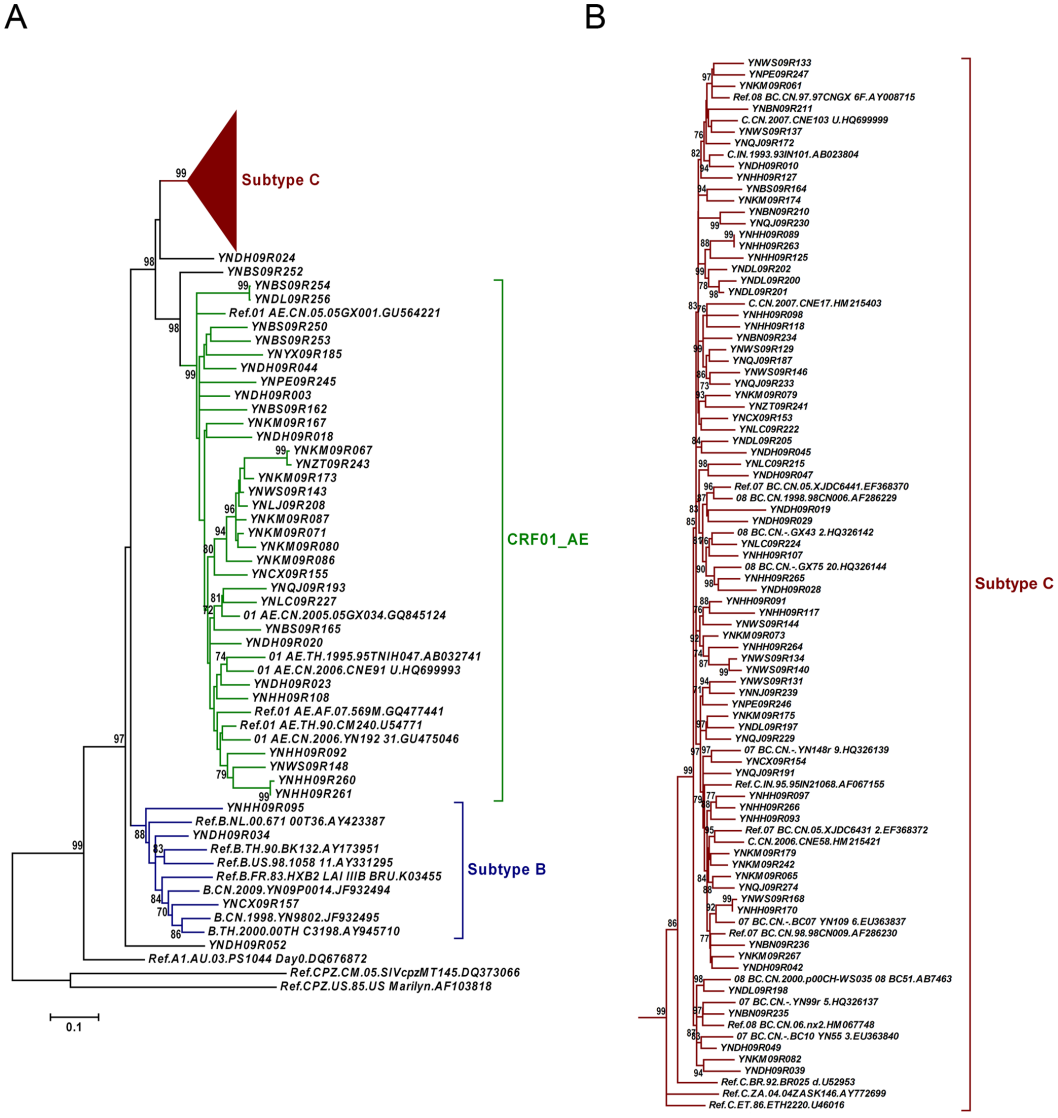
Neighbor-joining phylogenetic tree of partial *env* gene from recently infected individuals confirmed by BED-CEIA. A, Neighbor-joining phylogenetic tree for 108 *env* sequences and relative reference sequences. B, Subtype C cluster from the phylogenetic tree shown in A. The scale bar indicates 10% nucleotide sequence divergence. Values on the branches represent the percentage of 1000 bootstrap replicates and bootstrap values over 70% are shown in the tree.

**Figure 3:**
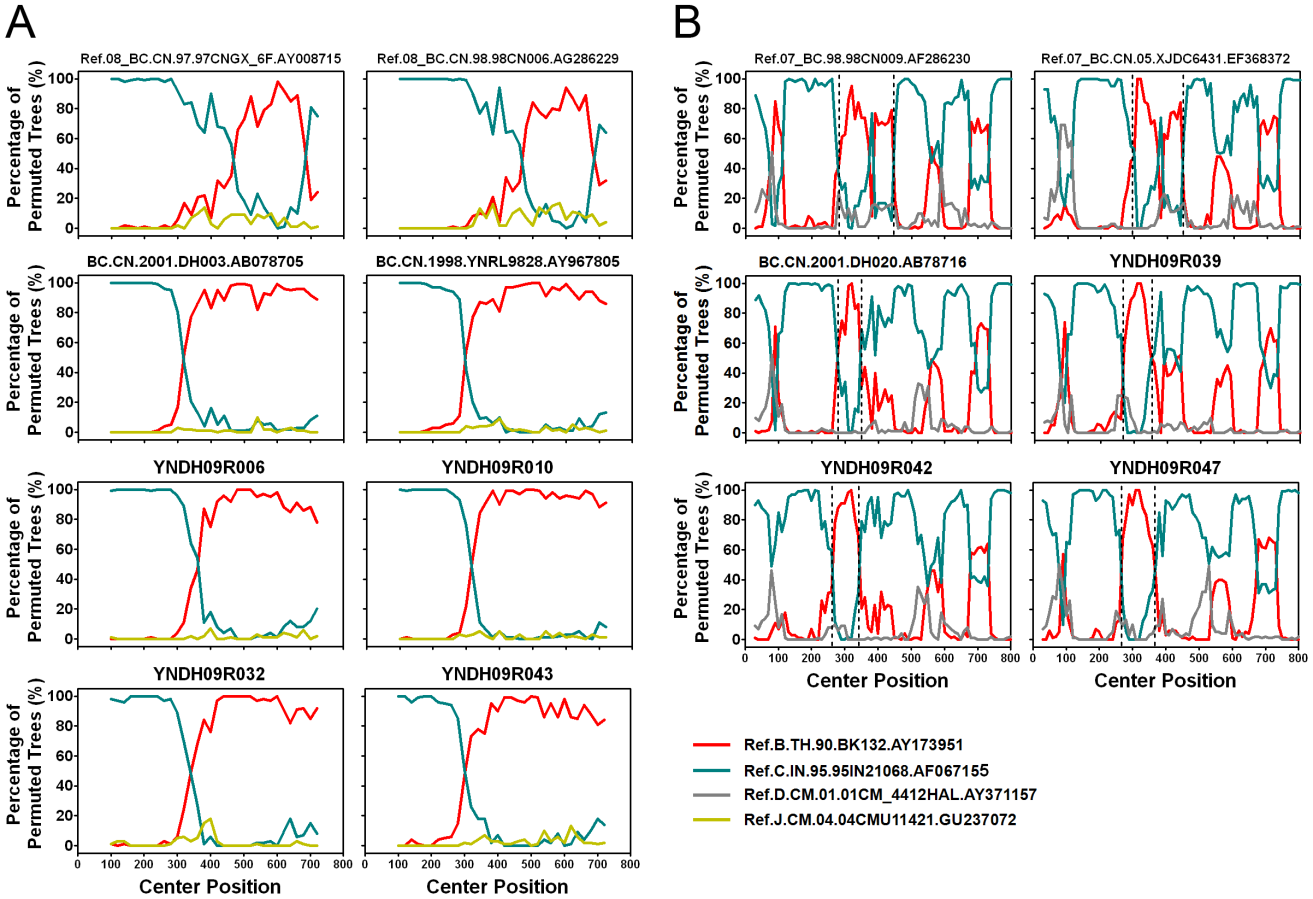
Bootscanning analysis of *gag* sequences of BC recombinants. A, Bootscanning analysis of reference sequences of CRF08_BC and representative sequences in BC Cluster 1 from the phylogenetic tree shown in Figure 1A. The conditions used for this analysis: Window: 200 bp, step: 20 bp, GapStrip: on, reps: 100, Kinura (2-parameter), T/t: 2.0. B, Bootscanning analysis of reference sequences of CRF07_BC and representative sequences in BC Cluster 2 from the phylogenetic tree shown in Figure 1A. The conditions used for this analysis: Window: 60 bp, Step: 10 bp, GapStrip: on, Reps: 100, Kinura (2-parameter), T/t: 2.0. The reference sequences are shown at the bottom right of the figure.

**Figure 4:**
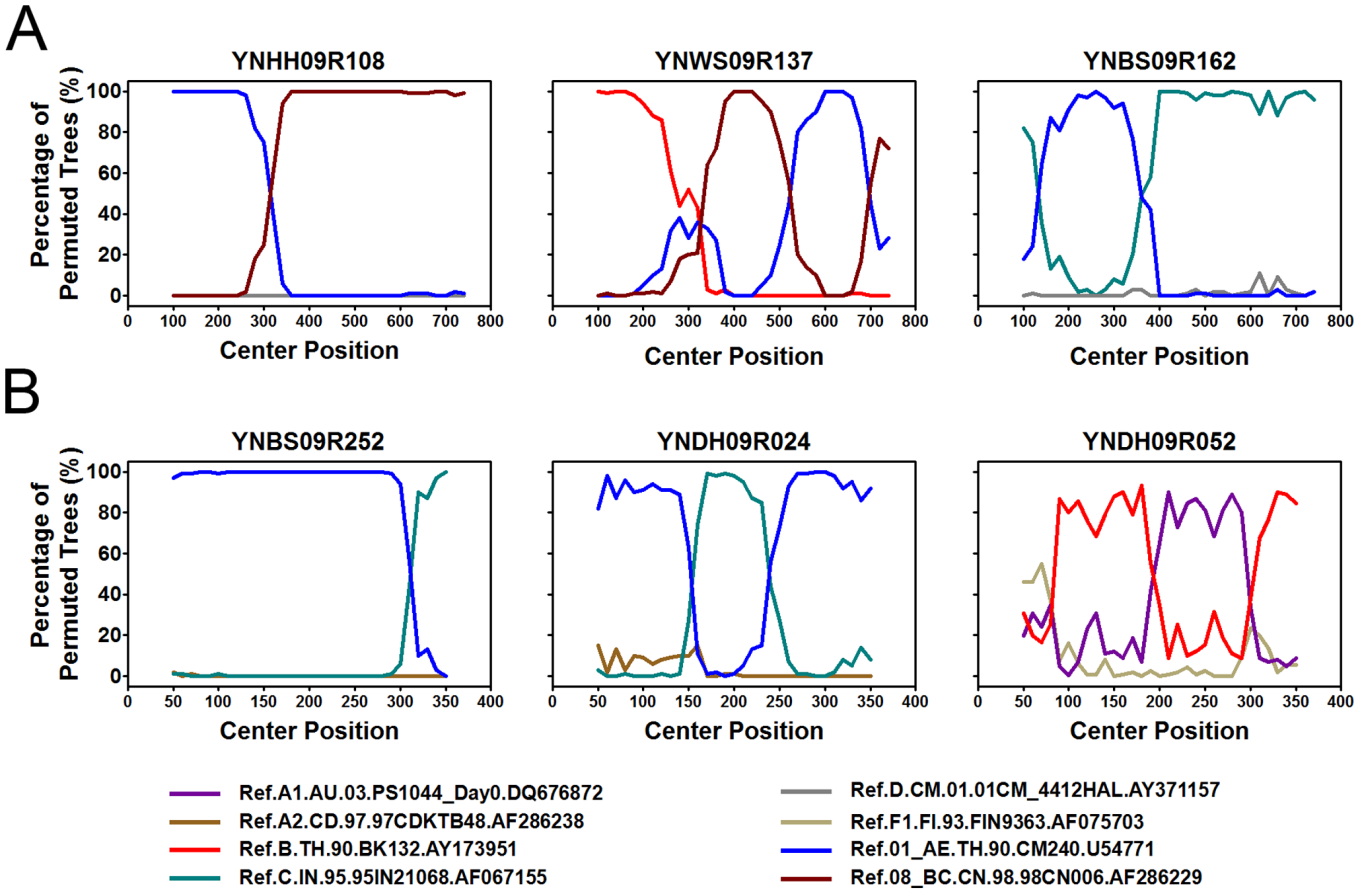
Bootscanning analysis of *gag* and *env* sequences of new recombination. A, Bootscanning analysis of *gag* sequences of YNHH09R108, YNBS09R162 and YNWS09R137. The conditions used for this analysis: Window: 200 bp, step: 20 bp, GapStrip: on, reps: 100, Kinura (2-parameter), T/t: 2.0. B, Bootscanning analysis of env sequences of YNBS09R252, YNDH09R024 and YNDH09R052. The conditions used for this analysis: Window: 100 bp, Step: 10 bp, GapStrip: on, Reps: 100, Kinura (2-parameter), T/t: 2.0. The reference sequences are shown at the bottom.

Phylogenetic analyses of 120 *gag* fragments (912 bp, encoding portions of p17 and p24), and *env* fragments (477 bp, encoding V3∼V4 region) were performed (Fig. 1 and Fig. 2). As Among 120 *gag* sequences, 59 (49.2%), 12 (10.0%), 3 (2.5%), 5 (4.2%) and 24 (20.0%) sequences were identified as CRF08_BC, CRF07_BC, subtype C, subtype B and CRF01_AE, respectively (Fig. 1). The remaining 17 sequences clustered between or outsite of the clades of subtypes/CRFs, suggesting that they might be intersubtype recombinations. To verify whether they are recombinants, bootscanning analyses using Simplot software were performed, which revealed 4 recombinant forms, including 14 BC, 1 CRF01_AE/CRF08_BC, 1 CRF01_AE/C and 1 B/CRF08_BC/CRF01_AE recombinants (Fig. 3 and Fig. 4A). Among BC recombinants, two discrete BC clusters were identified, in which the BC recombinants found previously in western Yunnan [11], [17] were also clustered (Fig. 1A). Furthermore, each BC cluster shared a similar recombination pattern in *gag* region (Fig. 3). This suggests that potential BC Circulating Recombinant Forms might circulate in this region. Phylogenetic and bootscanning analyses of *env* region revealed 31 (28.7%) CRF01_AE, 3 (2.8%) subtype B, 71 (65.7%) subtype C, 2 (1.9%) CRF01_AE/C and 1 (0.9%) A1/B (Fig. 2 and Fig. 4B).

By combining the phylogenetic tree analyses of *gag* and *env*, a total of 130 samples generated interpretable sequence data, revealing 2 subtypes, 3 CRFs and 7 discrete URFs. Among the recent infections, CRF08_BC was the most common genotype (40.8%, 53/130), followed by the URFs (27.7%, 36/130), CRF01_AE (18.5%, 24/130), CRF07_BC (9.2%, 12/130), subtype B (2.3%, 3/130) and subtype C (1.5%, 2/130) (Table 2).

**Table 2:** Demographic characteristics and subtypes of study subjects.

		Subjects	Genotypes							
			B	C	CRF01_AE	CRF07_BC	CRF08_BC	URFs	χ2	*P*
**Total**		130 (100.0%)	3 (2.3%)	2 (1.5%)	24 (18.5%)	12 (9.2%)	53 (40.8%)	36 (27.7%)		
**Gender**									7.373	0.161
Male		77 (59.2%)	2 (1.5%)	1 (0.8%)	13 (10.0%)	10 (7.7%)	26 (20.0%)	25 (19.2%)		
Female		53 (40.8%)	1 (0.8%)	1 (0.8%)	11 (8.5%)	2 (1.5%)	27 (20.8%)	11 (8.5%)		
**Age**									4.921	0.296^a^
≤20		9 (6.9%)	0 (0.0%)	0 (0.0%)	5 (3.8%)	0 (0.0%)	2 (1.5%)	2 (1.5%)		
21–30		52 (40.0%)	0 (0.0%)	0 (0.0%)	9 (6.9%)	6 (4.6%)	23 (17.7%)	14 (10.8%)		
31–40		39 (30.0%)	2 (1.5%)	1 (0.8%)	7 (5.4%)	4 (3.1%)	14 (10.8%)	12 (9.2%)		
41–50		13 (10.0%)	0 (0.0%)	1 (0.8%)	3 (2.3%)	1 (0.8%)	6 (4.6%)	2 (1.5%)		
≥51		17 (13.1%)	1 (0.8%)	0 (0.0%)	0 (0.0%)	1 (0.8%)	9 (6.9%)	6 (4.6%)		
**Race/Ethnicity**									11.886	0.022
Han		94 (72.3%)	0 (0.0%)	1 (0.8%)	21 (16.2%)	10 (7.7%)	39 (30.0%)	23 (17.7%)		
Others		36 (27.7%)	3 (2.3%)	1 (0.8%)	3 (2.3%)	2 (1.5%)	14 (10.8%)	13 (10.0%)		
**Marital Status**									10.692	0.317
Unmarried		42 (32.3%)	0 (0.0%)	0 (0.0%)	12 (9.2%)	5 (3.8%)	14 (10.8%)	11 (8.5%)		
Married		73 (56.2%)	2 (1.5%)	2 (1.5%)	11 (8.5%)	5 (3.8%)	34 (26.2%)	19 (14.6%)		
Divorced/Widowed		15 (11.5%)	1 (0.8%)	0 (0.0%)	1 (0.8%)	2 (1.5%)	5 (3.8%)	6 (4.6%)		
**Infection Routes**									6.940	0.173^b^
Heterosexual contact		100 (76.9%)	2 (1.5%)	1 (0.8%)	17 (13.1%)	9 (6.9%)	44 (33.8%)	27 (20.8%)		
Intravenous drug injection		20 (15.4%)	1 (0.8%)	1 (0.8%)	1 (0.8%)	3 (2.3%)	7 (5.4%)	7 (5.4%)		
Homosexual contact		3 (2.3%)	0 (0.0%)	0 (0.0%)	3 (2.3%)	0 (0.0%)	0 (0.0%)	0 (0.0%)		
Maternal-neonatal transmission		5 (3.8%)	0 (0.0%)	0 (0.0%)	3 (2.3%)	0 (0.0%)	0 (0.0%)	2 (1.5%)		
Unknown		2 (1.5%)	0 (0.0%)	0 (0.0%)	0 (0.0%)	0 (0.0%)	2 (1.5%)	0 (0.0%)		

a Non parametric Kruskal Wallis test was applied.

b Compared the constitutions of subtypes between heterosexual contact and intravenous drug injection.

Interestingly, the proportion of the URFs exceeded that of CRF01_AE and ranked second, which suggested that new recombination occurs frequently among the HIV-1 recently-infected population. Among the URFs, two types of recombination patterns existed. In pattern one, as mentioned above, mosaic construction was detected in *gag* or *env* region (Fig. 3 and Fig. 4). In pattern two, URFs’ *gag* and *env* region belonged to different subtypes or CRFs, respectively. As shown in Table 2 and Tabel 3, 69.4% (25/36) of the individuals with URFs were male, and 27 individuals with URFs (75.0%) were infected through heterosexual contact, while seven (19.4%) through intravenous drug use and two (5.6%) through maternal-neonatal transmission. Furthermore, CRF01_AE was involved in 55.5% (20/36) of the URFs (Table 3). Among all the URFs, BC recombinants were the most common recombinant form (41.7%, 15/36); the other six discrete URFs included CRF01_AE/C (27.8%, 10/36), CRF08_BC/CRF01_AE (19.4%, 7/36), CRF01_AE/B (2.8%, 1/36), CRF01_AE/BC (2.8%, 1/36), B/CRF08_BC/CRF01_AE (2.8%, 1/36) and A1/B (2.8%, 1/36). Since subtype C, CRF07_BC and CRF08_BC cannot be distinguished in the *env* region, the CRF01_AE/C group may include the following recombinants: CRF01_AE/C, CRF01_AE/CRF08_BC, and CRF01_AE/CRF07_BC.

**Table 3:** Demographic and genetic characteristics of 28 individuals with new recombinants.

Case Number	Prefecture	Sex	Age	Transmission Route	*gag*	*env*	URFs
YNDH09R006	Dehong	Male	23	Intravenous drug injection	BC	\	BC
YNDH09R010	Dehong	Male	36	Intravenous drug injection	BC	C	BC
YNDH09R011	Dehong	Male	37	Heterosexual contact	BC	\	BC
YNDH09R016	Dehong	Female	23	Heterosexual contact	BC	\	BC
YNDH09R027	Dehong	Male	6	Maternal-neonatal transmission	BC	\	BC
YNDH09R028	Dehong	Female	21	Heterosexual contact	BC	C	BC
YNDH09R032	Dehong	Female	44	Heterosexual contact	BC	\	BC
YNDH09R039	Dehong	Female	23	Heterosexual contact	BC	C	BC
YNDH09R042	Dehong	Male	23	Intravenous drug injection	BC	C	BC
YNDH09R043	Dehong	Male	11	Maternal-neonatal transmission	BC	\	BC
YNDH09R045	Dehong	Female	31	Heterosexual contact	BC	C	BC
YNDH09R047	Dehong	Male	32	Heterosexual contact	BC	C	BC
YNDH09R049	Dehong	Female	37	Heterosexual contact	B	C	BC
YNHH09R091	Honghe	Female	26	Heterosexual contact	BC	C	BC
YNHH09R100	Honghe	Male	38	Intravenous drug injection	BC	\	BC
YNBS09R162	Baoshan	Male	33	Heterosexual contact	CRF01_AE/C	CRF01_AE	CRF01_AE/C
YNBS09R164	Baoshan	Male	45	Heterosexual contact	CRF01_AE	C	CRF01_AE/C
YNBS09R253	Baoshan	Male	34	Heterosexual contact	C	CRF01_AE	CRF01_AE/C
YNDL09R200	Dali	Male	63	Heterosexual contact	CRF01_AE	C	CRF01_AE/C
YNDH09R024	Dehong	Male	22	Intravenous drug injection	\	CRF01_AE/C	CRF01_AE/C
YNSCDC09R172	Qujing	Male	27	Heterosexual contact	CRF01_AE	C	CRF01_AE/C
YNQJ09R191	Qujing	Female	23	Heterosexual contact	CRF01_AE	C	CRF01_AE/C
YNQJ09R229	Qujing	Male	30	Intravenous drug injection	CRF01_AE	C	CRF01_AE/C
YNBN09R211	Xishuangbanna	Female	61	Heterosexual contact	CRF01_AE	C	CRF01_AE/C
YNBN09R234	Xishuangbanna	Male	54	Heterosexual contact	CRF01_AE	C	CRF01_AE/C
YNCX09R155	Chuxiong	Female	27	Heterosexual contact	CRF08_BC	CRF01_AE	CRF08_BC/CRF01_AE
YNHH09R108	Honghe	Male	21	Heterosexual contact	CRF01_AE/CRF08_BC	CRF01_AE	CRF08_BC/CRF01_AE
YNSCDC09R173	Kunming	Male	57	Heterosexual contact	CRF08_BC	CRF01_AE	CRF08_BC/CRF01_AE
YNLJ09R208	Lijiang	Male	22	Heterosexual contact	CRF08_BC	CRF01_AE	CRF08_BC/CRF01_AE
YNQJ09R193	Qujing	Male	38	Intravenous drug injection	CRF08_BC	CRF01_AE	CRF08_BC/CRF01_AE
YNYX09R185	Yuxi	Male	76	Heterosexual contact	CRF08_BC	CRF01_AE	CRF08_BC/CRF01_AE
YNZT09R243	Zhaotong	Male	40	Heterosexual contact	CRF08_BC	CRF01_AE	CRF08_BC/CRF01_AE
YNCX09R157	Chuxiong	Male	23	Heterosexual contact	CRF01_AE	B	CRF01_AE/B
YNBS09R252	Baoshan	Female	34	Heterosexual contact	B	CRF01_AE/C	CRF01_AE/BC
YNDH09R052	Dehong	Male	60	Heterosexual contact	\	A1/B	A1/B
YNWS09R137	Wenshan	Male	36	Heterosexual contact	B/CRF08_BC/CRF01_AE	C	B/CRF08_BC/CRF01_AE

### Distribution Characteristic of HIV-1 Genotypes by Infection Routes

To better characterize the distribution of HIV-1 genotype, we performed a detailed demographic study. The distribution of genotypes by gender, age, or marital status of patients showed no significant differences (Table 2). However, the distribution of genotypes by ethnicity showed statistical difference, mainly because subtype B was confined to Dai and Yi ethnicities. We found intermixing of subtypes/CRFs in different infection routes. In the heterosexually transmitted population, CRF08_BC accounted for 44.0% (44/100), URFs for 27.0% (27/100), CRF01_AE for 17.0% (17/100), CRF07_BC for 9.0% (9/100), subtype B for 2.0% (2/100), and subtype C 1.0% (1/100). In IDUs, CRF08_BC accounted for 35.0% (7/20), URFs for 35.0% (7/20), CRF07_BC for 15.0% (3/20), subtype C for 5.0% (1/20), CRF01_AE for 5.0% (1/20), and subtype B for 5.0% (1/20). These data showed that CRF08_BC has become the predominant viral genotype both in heterosexuals and IDUs. Further, genotypic distribution showed no statistical difference in these two populations (Table 2), suggesting that genotypes tend to randomly distribute among patients infected through heterosexual contact and intravenous drug use, and the separation of subtype/CRF by different risk groups appears to have diminished. Otherwise, three CRF01_AE strains and two URFs were detected in maternal-neonatal transmission, and three CRF01_AE strains in the homosexually transmitted population (Table 2). Finally, the distribution of different types of URFs in different infection routes showed no statistical difference (Table 4).

**Table 4:** The distribution of URFs in different transmission routes.

Infection Routes	BC	CRF01_AE/C	CRF08_BC/CRF01_AE	CRF01_AE/B	CRF01_AE/BC	B/CRF08_BC/CRF01_AE	A1/B	Total	χ2	*P*
Heterosexual contact	9	8	6	1	1	1	1	27	10.023	0.912
Intravenous drug injection	4	2	1	0	0	0	0	7		
Maternal-neonataltransmission	2	0	0	0	0	0	0	2		
Total	15	10	7	1	1	1	1	36		

### Geographic Distribution Characteristic of HIV-1 Genotypes

**Figure 5:**
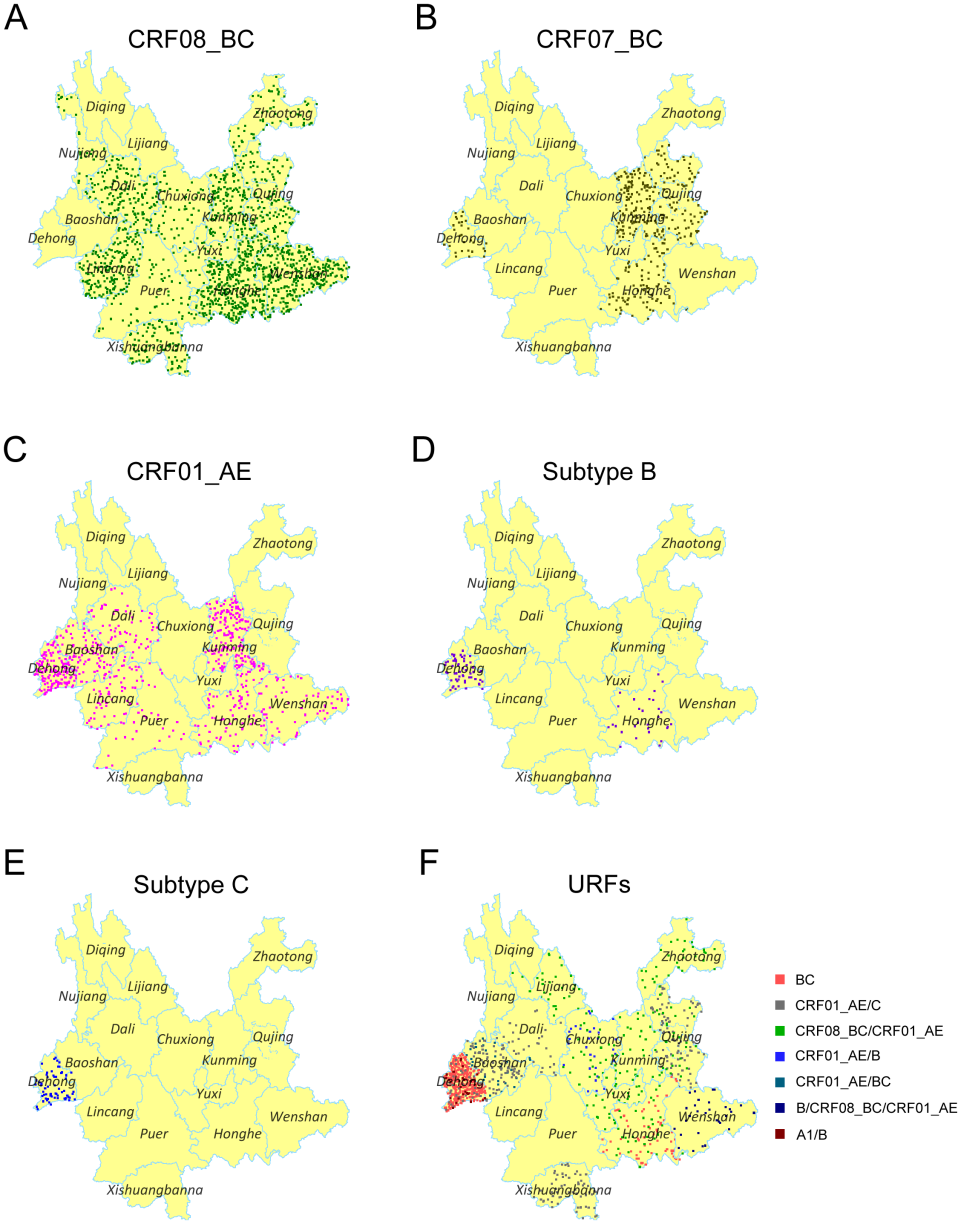
Geographic distribution of the main genotypes in Yunnan. A, B, C, D, E and F are Dot Density Map for CRF08_BC, CRF07_BC, CRF01_AE, subtype B, subtype C and URFs, respectively, which showed the percentage of each genotype in each prefecture. One dot was defined as 0.025% of the population.

Finally we analyzed the geographic distribution of each HIV-1 genotypes. CRF08_BC was distributed almost throughout the whole province, except Baoshan, Lijiang and Diqing (Fig. 5A). In line with the previous report, this viral genotype predominated in east Yunnan, including 3 highly affected prefectures: Wenshan, Honghe and Kunming. Additionally, the prevalence of CRF08_BC increased in west Yunnan, especially in Dali and Lincang. Conversely, CRF07_BC was mainly distributed in the eastern region including Honghe, Kunming and Qujing (Fig. 5B). Previously, CRF01_AE was limited to the western prefectures (Dehong, Baoshan and Lincang). In this study, we found that CRF01_AE had spread to the eastern prefectures (Kunming, Honghe and Wenshan), and predominated in Kunming, the capital of Yunnan (Fig. 5C). Traditionally, subtypes B and C were confined in Dehong where these two subtypes were first introduced into Yunnan in the late 1980s and early 1990s. Here, we found that subtype B and C still predominated in Dehong, and separate subtype B strains were found in Honghe (Fig. 5D and 5E). The 36 URFs distributed widely, and were in 12 prefectures (Fig. 5F). There were five prefectures with more than one type of URF detected (Dehong, Baoshan, Chuxiong, Honghe and Qujing). In line with the distribution of subtype B and C, BC recombinants predominated in Dehong, while CRF01_AE/C and CRF08_BC/CRF01_AE were not limited to the local area, but were scattered widely.

**Figure 6:**
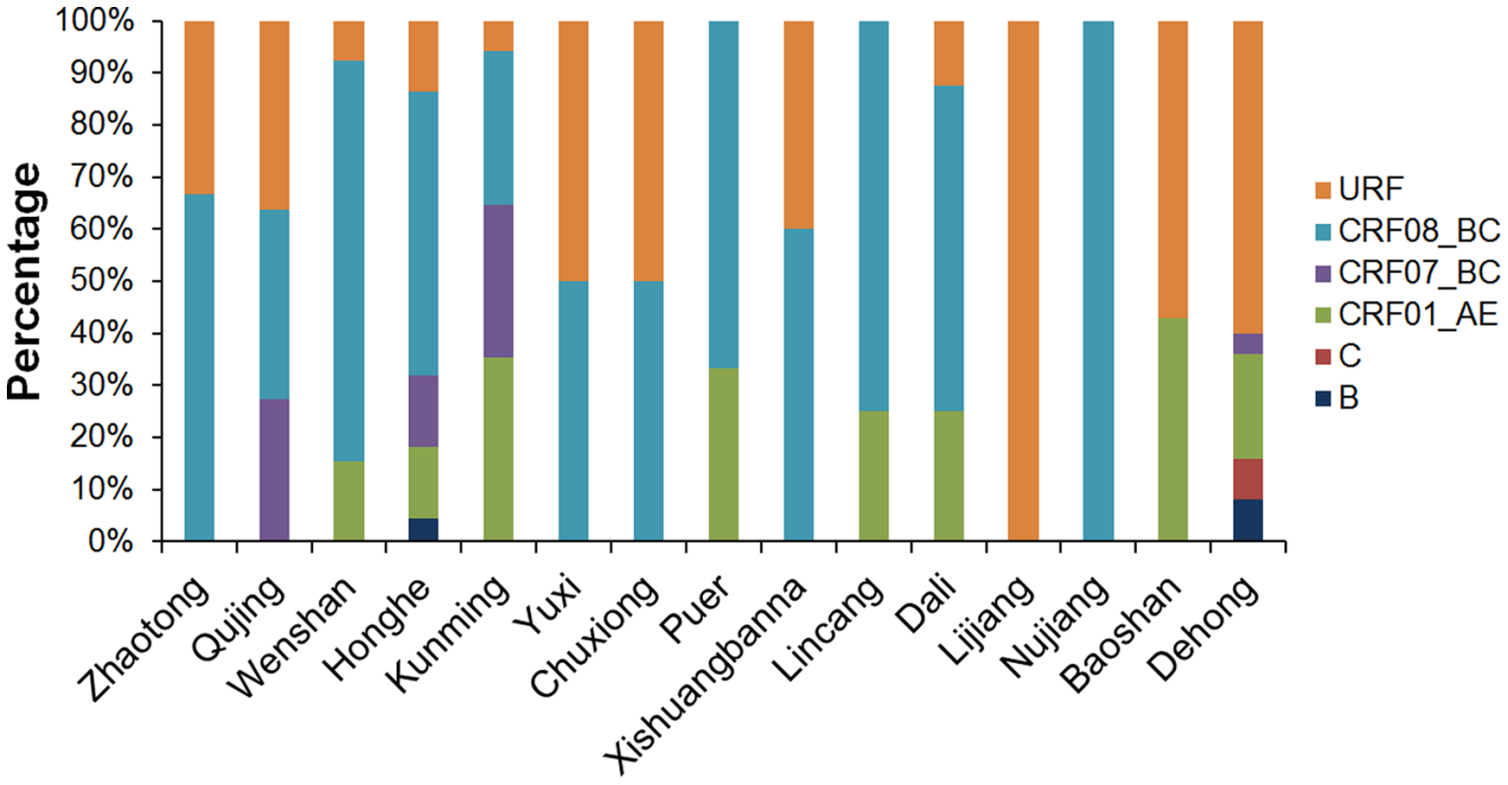
The Distribution of HIV-1 subtypes/CRFs for each prefecture in Yunnan.

Except for the three northwest prefectures, Nujiang, Lijiang and Diqing (HIV-1 genetic analysis in Diqing was missing because no recent infector was detected in Diqing), the other 13 prefectures have more than one viral genotype present, and Dehong, Honghe and Kunming showed more complex genetic diversity (Fig. 6).

## Discussion

In the present work, we conducted an HIV-1 molecular epidemiological study to disclose new trends in recently-infected individuals of Yunnan Province, the province most severely affected by HIV/AIDS in China. By analyzing the HIV-1 genes *gag* and *env*, we described the distribution characteristic of HIV-1 subtypes, CRFs and URFs in this area. We found that CRF08_BC was the most common CRF, which predominated in both east and west Yunnan among IDUs and the sexually transmitted population. Exceeding the frequency of CRF01_AE, URFs became the second most common HIV-1 strain which suggests continuous risk behavior still exists among certain groups. Importantly, the distribution of these genotypes was not closely associated with transmission route.

By the end of 2011, the cumulative number of reported HIV/AIDS in Yunnan was 95296, accounting for 21.0% of the total national figure. Yunnan has shown more diverse HIV-1 genetics than any other HIV-1 high-prevalent province in China, including Guangxi, Sichuan, Xijiang, Guangdong and Hennan [22], [23]. The early reports on characterization of HIV-1 genotypes in Yunnan were confined to the IDU population, which revealed multiple HIV-1 genotypes, including B, B’, C, CRF01_AE, CRF07_BC, CRF08_BC and URF [2], [3], [5], [9], [11], [16], [17], [24]. During the transition of main transmission route from intravenous injection to sexual contact, diverse HIV-1 strains were also identified among individuals who acquired HIV-1 through sexual contact [15], [16], [24]. Additionally, phylogenetic analysis showed that sequences from IDUs intermingled with those from individuals infected through sexual contact within each subtype of HIV-1 [24], which suggested HIV-1 strains circulating in the sexually transmitted population might come from those in IDUs through commercial or non commercial sex contacts.

In the early 2000s, CRF08_BC and CRF01_AE were dominant in IDUs and those with sexually transmitted infections, respectively [1], [16]. At that time, it was presumed that the prevalence of CRF01_AE would increase with sexual transmission rising [16]. However, in recently infected people, we found that CRF08_BC predominated not only in IDUs, but also in the heterosexually transmitted population. The influx of CRF08_BC into the heterosexually transmitted population may occur through male IDUs who visit female sex workers (FSWs) as well as FSWs who inject drugs. These groups have played a crucial role in the transition of the epidemic from being primarily IDU-driven to sexually driven [25]. Another possible reason is the transmission from HIV-1 positive IDUs to their spouses and regular sexual partners through unprotected sex. Furthermore, the distributions of the main HIV-1 genetic strains showed no statistical differences between IDUs and the heterosexually transmitted population. This suggests that these strains tended to randomly distribute into these two populations, and the phenomenon observed in previous studies [15], [16] of different subtypes/CRF predominating in different risk groups appears to have diminished. The changing characteristics of HIV-1 molecular epidemiology suggest that the bridging population is still the primary force behind the development of the HIV-1 epidemic in Yunnan. Thus, measures for effective epidemic control should be further developed and performed among the high-risk groups, particularly IDUs, commercial sex workers and the spouses of HIV-1 positive individuals. Key strategies should include behavior intervention, scaling up identification of HIV-1 infected persons among the high risk groups, and providing antiretroviral therapy (ART) to them to effectively reduce HIV-1 incidence by decreasing the viral load level.

Recombination between HIV-1 genotypes is an important mechanism that contributes substantially to the genetic complexity of HIV-1 and may result in establishing epidemiologically important founder strains. The coexistence of multiple genotypes in the same area always causes the formation of CRFs. Typically, CRF07_BC and CRF08_BC have been thought to originate among IDUs in Yunnan [11], [12], but how this occurred was not well-understood. This past lack of knowledge may have been the result of the use of the *env* region only in earlier studies [2], [6]. CRF07_BC and CRF08_BC are composed mostly of subtype C and contain a few small segments of subtype B’. Thus, distinguishing between CRF07_BC/CRF08_BC and subtype C based on the *env* region alone would have been difficult. It has been reported that the nearly full-length genome analyses of the *env*-based subtype C samples revealed that they were actually BC recombinants [11]. In this study, we found two discrete BC clusters. The sequences in these two clades also clustered together with those of the BC recombinants detected in western Yunnan in the late 1990s and early 2000s [11], [17]. Furthermore, each cluster has its own recombination pattern in *gag* region, which is distinct from those of CRF07_BC and CRF08_BC. All of this suggests that new CRFs_BC might have been established in this area. To prove this hypothesis, the complete sequencing of full-length HIV-1 genomes is required in future research.

Besides CRFs, another striking result is that diverse URFs arise continually. URFs reported before mainly included new BC recombinants, CRF07_BC/CRF08_BC recombinants, and C/CRF01_AE recombinants [11], [16], [17], [26], [27], [28], [29], most of which were found among IDUs. In this work, we found that the types and quantity of URFs increased among recent infections. The frequency of URFs was exceeded only by that of CRF08_BC. Of the seven types of URFs detected, nearly 70% were identified among heterosexually-infected individuals, and 55.5% were CRF01_AE relative URFs. In Yunnan, CRF01_AE was first found among FSWs [7], and was the preponderant strain in this population in the early stage of the HIV-1 epidemic [1], [16]. The high proportion of CRF01_AE relative URFs suggests that active recombination took place through commercial sex behavior. The geographic distribution of URFs is growing and covered 12 out of the 16 prefectures. All of these contributed substantially to the genetic complexity of HIV-1 in Yunnan. The formation and linkage of these URFs will be our research focus in the future, which will elucidate the development of the HIV-1 epidemic of Yunnan, and provide references for HIV control in this area.

Furthermore, the HIV-1 strains showed different temporal and spatial dynamics in the process of spreading. We found that CRF07_BC and CRF08_BC were both highly prevalent in east Yunnan, but CRF08_BC was distributed more widely than CRF07_BC, covering almost the entire province. Presently, we do not know whether the transmission capacities of these two CRFs differ. CRF01_AE spread to east Yunnan, particularly in Kunming where the proportion of CRF01_AE remained at a low level in the early 2000s [30]. Thus, a general trend was that HIV-1 strains transferred from their original highly-prevalent area to the rest of the province. The specificity and dynamics of HIV-1 transmission also resulted in the different distribution of HIV-1 genotypes in different areas. For example, Dehong and Honghe showed the most complicated HIV-1 genetic diversity in the east and the west, respectively. However, their distribution patterns were totally different. CRF08_BC was predominant in Honghe, while BC recombinants and subtype C mainly circulated in Dehong. On the other hand, the neighboring prefectures shared similar distribution patterns, such as Honghe and Wenshan, Yuxi and Chuxiong, Dali and Lincang.

Since the first acknowledged HIV-1 epidemic in China began in Yunnan, this region has become a critical area bridging HIV-1 epidemics in southeast Asia with the subsequent inland epidemic. Through durg trafficking, some genotypes originally found in Yunnan had spread not just to the neighboring provinces of Guangxi (CRF08_BC) [12], [31], [32], [33], [34] and Sichuan (CRF07_BC) [35], but also to the north-western province of Xijiang (CRF07_BC) [12], [35] and the central province of Henan (subtype B’) [36]. On the other hand, the countries bordering Yunnan display their own characteristics of HIV-1 molecular epidemiology. In Vietnam, CRF01_AE is the predominant strain [37]. However, in Myanmar, besides B’, C and CRF01_AE, recombinant forms between B’, C and CRF01_AE are increasing dramatically [38]. A similar trend is seen in Dehong, where the proportion of URFs is higher than any other subtype/CRF. These suggest that the China-Myanmar border area constitutes a hot spot of active recombination in Asia. The bidirectional transmission of HIV-1 between Yunnan and neighboring countries means that the epidemic of Yunnan could potentially influence neighboring countries. All of this suggests that the main strains prevailing in China and neighboring countries could be found in Yunnan. Thus, comprehensively understanding HIV-1 molecular epidemical characteristics in Yunnan plays an important role in efficient control of the HIV-1 epidemic, as well as vaccine design and evaluation for China and the neighboring countries.

One limitation of this work was the relatively low PCR amplification rate (*gag*: 39.0%, 120/308; *env*: 35.1%, 108/308), which also occurrd in our previous work [16]. The reasons may be RNA degradation owing to poor serum storage and transportation conditions. In fact, the plasma specimens had been used for HIV-1 screening, confirmation and BED-CEIA before genetic analysis was performed, during which time the samples underwent several freeze-thaw cycles. Other reasons may include low viral load in some cases and sequence variations at the primer binding sites. The low amplification rate meant that some low-frequent viral genotype might be missed, however it is unlikely to compromise the interpretation of the general genetic distribution. This is because the constituent ratios of the 130 subjects with definite genotyping and the total of 308 recently HIV-infected individuals showed no statistical differences by area of source, gender, age, ethnicity, marital status and infection routes.

In summary, we found that six HIV-1 genotypes circulated in the recently infected individuals of Yunnan, and the distribution of the main genotypes was not closely associated with transmission route. HIV-1 genetics became more diverse because of frequent intersubtypes/CRFs recombinations. These findings showed new trends of HIV-1 molecular epidemiology with dynamic changes in the HIV-1 epidemic and will contribute to a better understanding of the distribution and evolution of HIV-1 in Yunnan as well as help to establish and modify public health efforts to prevent new infections.
